# Association of Cardiac Rehabilitation With All-Cause Mortality Among Patients With Cardiovascular Disease in the Netherlands

**DOI:** 10.1001/jamanetworkopen.2020.11686

**Published:** 2020-07-27

**Authors:** Thijs M. H. Eijsvogels, Martijn F. H. Maessen, Esmée A. Bakker, Esther P. Meindersma, Niels van Gorp, Nicole Pijnenburg, Paul D. Thompson, Maria T. E. Hopman

**Affiliations:** 1 Department of Physiology, Radboud Institute for Health Sciences, Radboud University Medical Centre, Nijmegen, the Netherlands; 2 Coöperatie Volksgezondheidszorg, Business Intelligence Services, Arnhem, the Netherlands; 3 Research Institute for Sports and Exercise Sciences, Liverpool John Moores University, Liverpool, United Kingdom; 4 Department of Cardiology, Radboud Institute for Health Sciences, Radboud University Medical Centre, Nijmegen, the Netherlands; 5 Division of Cardiology, Hartford Hospital, Hartford, Connecticut

## Abstract

**Importance:**

Cardiac rehabilitation (CR) is an effective strategy to improve clinical outcomes, but it remains underused in some subgroups of patients with cardiovascular disease (CVD).

**Objective:**

To investigate the implications of sex, age, socioeconomic status, CVD diagnosis, cardiothoracic surgery, and comorbidity for the association between CR participation and all-cause mortality.

**Design, Setting, and Participants:**

Observational cohort study with patient enrollment between July 1, 2012, and December 31, 2017, and a follow-up to March 19, 2020. The dates of analysis were March to May 2020. This study was performed among Dutch patients with CVD with a multidisciplinary outpatient CR program indication and who were insured at Coöperatie Volksgezondheidszorg, one of the largest health insurance companies in the Netherlands. Among 4.1 million beneficiaries, patients with CVD with an acute coronary event (myocardial infarction or unstable angina pectoris), stable angina pectoris, chronic heart failure, or cardiothoracic surgery (coronary artery bypass grafting, valve replacement, or percutaneous coronary intervention) were identified by inpatient diagnosis codes and included in the study.

**Main Outcomes and Measures:**

Cox proportional hazards models were used to evaluate the association between CR participation and all-cause mortality. Stabilized inverse propensity score weighting was used to account for patient and disease characteristics associated with CR participation.

**Results:**

Among 83 687 eligible patients with CVD (mean [SD] age, 67 [12] years; 60.4% [n = 50 512] men), only 31.3% (n = 26 171) participated in CR, with large variation across different subgroups (range, 5.1%-73.0%). During a mean (SD) of 4.7 (1.8) years of follow-up, 1966 CR participants (7.5%) and 13 443 CR nonparticipants (23.4%) died. After multivariable adjustment, CR participation was associated with a 32% lower risk of all-cause mortality (adjusted hazard ratio, 0.68; 95% CI, 0.65-0.71) compared with nonparticipation. Sex, age, socioeconomic status, and comorbidity did not alter risk reduction after CR participation, but a statistically significant interaction association was found across categories of CVD diagnosis and cardiothoracic surgery. Larger reductions in risk estimates for all-cause mortality were found after CR participation for STEMI (adjusted HR, 0.59; 95% CI, 0.52-0.68 vs 0.72; 95% CI, 0.65-0.79; *P* < .001), NSTEMI (adjusted HR, 0.64; 95% CI, 0.58-0.70 vs 0.72; 95% CI, 0.65-0.79; *P* < .001), and stable AP (adjusted HR, 0.69; 95% CI, 0.63-0.76 vs 0.72; 95% CI, 0.65-0.79; *P* < .001) compared with patients with chronic heart failure, whereas unstable AP had a smaller risk reduction (adjusted HR, 0.75; 95% CI, 0.67-0.85 vs 0.72; 95% CI, 0.65-0.79; *P* < .001).

**Conclusions and Relevance:**

In this cohort study, CR participation was associated with a 32% risk reduction in all-cause mortality, and this benefit was independent of sex, age, socioeconomic status, and comorbidity. These findings reinforce the importance of CR participation in secondary prevention and highlight the possibility that CR should be prescribed more widely to vulnerable patients with CVD, such as older adults with chronic diseases or multimorbidity.

## Key Points

**Question:**

What is the patient-specific, disease-specific, and comorbidity-specific association of cardiac rehabilitation participation with all-cause mortality?

**Findings:**

In this cohort study, 31% of 83 687 eligible patients with cardiovascular disease participated in a multidisciplinary outpatient cardiac rehabilitation program. Cardiac rehabilitation participation was associated with a 32% lower risk of all-cause mortality compared with nonparticipation, which was independent of patient-related and comorbidity-related characteristics.

**Meaning:**

Cardiac rehabilitation participation is associated with a lower mortality risk compared with nonparticipation; however, cardiac rehabilitation remains underused, especially in older adults with chronic diseases or multimorbidity.

## Teaser

This cohort study investigates the implications of sex, age, socioeconomic status, cardiovascular disease (CVD) diagnosis, cardiothoracic surgery, and comorbidity for the association between cardiac rehabilitation participation and all-cause mortality.

## Introduction

Cardiac rehabilitation (CR) is a key component of secondary prevention strategies for patients with cardiovascular disease (CVD).^1^ Risk reduction for all-cause mortality, CVD mortality, unplanned hospitalization, and reinfarction has been reported in CR participants vs nonparticipants in multiple meta-analyses.^2,3,4,5,6^ Therefore, the American Heart Association, American College of Cardiology, and European Society of Cardiology have included CR referral as a Class IA recommendation for stable patients after an acute coronary syndrome or heart failure diagnosis.^7,8,9,10^ Despite these strong recommendations, participation rates of patients with CVD among hospital-based CR programs remain low, varying between 10% and 35% of eligible patients.^11,12^ Demographic and clinical factors, such as female sex, advanced age, socioeconomic status (SES), recent cardiothoracic surgery, and existing comorbidity, are known to reduce CR referral and participation,^13,14^ but evidence to refute these referral biases is scarce.

The present study aimed to investigate the implications of sex, age, SES, CVD diagnosis, cardiothoracic surgery, and comorbidity for the association between participation in a multidisciplinary outpatient CR program and all-cause mortality risk reduction. The study analyzed health insurance claims data from a nationwide cohort of 4.1 million individuals in the Netherlands. We hypothesized that participation in CR would be associated with a lower mortality risk for patients with CVD compared with nonparticipation, and we expected the risk reduction would vary across subgroups with different risk profiles.

## Methods

### Cohort Characteristics and Patient Selection

In the Netherlands, it is mandatory to have health insurance. For the present study, health insurance claims data were used from Dutch patients with CVD with a multidisciplinary outpatient CR program indication and who were insured at Coöperatie Volksgezondheidszorg (VGZ), one of the largest health insurance companies in the Netherlands. The VGZ cohort consists of 4.1 million beneficiaries, representing 24% of the Dutch population,^15^ with nationwide coverage and a representative sample of individuals from all age categories, SES, and both rural and urban areas (eFigure 1 in the Supplement). Individuals eligible for the present study were patients with a new diagnosis of CVD with an indication for CR according to Dutch guidelines,^16^ including an acute coronary event (myocardial infarction [MI] or unstable angina pectoris [AP]), stable AP, chronic heart failure, or cardiothoracic surgery (coronary artery bypass grafting [CABG], valve replacement, or percutaneous coronary intervention [PCI]). Reimbursement for outpatient CR is provided on the condition that the patient is referred by a cardiologist. Health insurance claims data were used to assess the type and date of diagnosis, and patients were enrolled between July 1, 2012, and December 31, 2017, with follow-up to March 19, 2020. The dates of analysis were March to May 2020. This study followed the Strengthening the Reporting of Observational Studies in Epidemiology (STROBE) reporting guideline. The Radboud University Medical Center Institutional Review Board deemed that this observational cohort study was exempt from informed consent because it involved retrospective analysis of an anonymized data set.

### CR in the Netherlands

The Dutch Society of Cardiology released a multidisciplinary CR guideline,^16^ which is consistent with international recommendations.^17,18^ Group-based outpatient CR programs consist of the following 4 modules: (1) supervised exercise training, (2) mental health and stress relief, (3) social health, and (4) cardiovascular risk management. The multidisciplinary CR program typically lasts 6 to 12 weeks; on average, 85% of the patients receive exercise training, 39% receive relaxation therapy, 17% receive lifestyle modification therapy, and 75% receive education.^19^ Hence, the content of the contemporary Dutch CR program is largely comparable to that of CR programs in other countries. Accessibility to CR in the Netherlands is excellent because most CR facilities are located near the residence of patients (<30 km).

### Study Population and Analytic Cohort

A total of 101 940 patients met the initial selection criteria. Consistent with previous observational studies,^20,21^ patients were classified as having received CR if a claim was filed for at least one of the group-based outpatient treatments. Excluded from further analysis were patients who (1) were insured less than 365 days before or less than 180 days after their diagnosis of CVD, (2) began CR more than 90 days after their diagnosis, or (3) died within 180 days after their diagnosis. Patients with multiple CVD-related insurance claims within 30 days after the initial diagnosis were reclassified according to the most severe diagnosis using the following rankings for CVD diagnosis and cardiothoracic surgery.^20^ For CVD diagnosis, the ranking was (1) ST-segment elevation MI (STEMI), (2) non-STEMI (NSTEMI), (3) unstable AP,(4) stable AP, and (5) chronic heart failure. For cardiothoracic surgery, the ranking was (1) CABG, (2) valve replacement, (3) acute PCI, and (4) elective PCI. Accordingly, 83 687 eligible patients with CVD were available for analysis (eFigure 2 in the Supplement).

### Primary Outcome

Survival status of study participants was obtained from the Dutch Population Register, and the date of death was extracted if applicable. All-cause mortality was the primary outcome, and survival time in days was calculated from the date of the initial diagnosis of CVD. The maximum follow-up in the cohort was 7.7 years (92 months). Patients were censored if they switched to another health insurance company or if they were still alive during the last survival check.

### Patient Characteristics

Sex and age were extracted from the VGZ health insurance database. Income and SES were obtained from the national statistical office (Statistics Netherlands) using postal code information for the patient’s residence. Socioeconomic status was classified using the SES score from Statistics Netherlands as low (bottom 40%), moderate (middle 30%), or high (upper 30%) (eFigure 1C in the Supplement).

### Comorbidity

Comorbidity was identified using diagnosis codes from the Dutch Business Intelligence Center for Healthcare (Vektis). Insurance claims data for pharmaceutical agents were used to evaluate the association of diabetes, cancer, gout, Parkinson disease, respiratory diseases, thyroid diseases, and dementia as binary variables with CR outcomes.^22^ We evaluated the consequences of multimorbidity using the Charlson Comorbidity Index (CCI). As a continuous variable, the CCI was calculated as the sum of comorbid conditions^23^ as follows. One point was given for MI, congestive heart failure, peripheral vascular disease, cerebrovascular disease, dementia, chronic pulmonary disease, ulcer disease, mild liver disease, and diabetes. Two points were given for diabetes with end-organ damage, any tumor, leukemia, and lymphoma. Three points were given for moderate to severe liver disease. Six points were given for metastatic solid tumors and AIDS. We compared the association of CR with all-cause mortality across patients with CCI scores of 1 or less, 2, 3, 4, and 5 or greater.

### Statistical Analysis

Baseline characteristics were summarized as mean (SD) or median (interquartile range) for continuous variables and as number (percentage) for categorical variables. Differences in individual and disease characteristics between CR participants and nonparticipants were assessed using *t* test for continuous variables and χ^2^ test for categorical variables. Kaplan-Meier curves and a log-rank test were used to assess the difference in all-cause mortality between CR participants and nonparticipants.

Information about patient characteristics, income, SES, distance to CR facility, disease characteristics, comorbidity, pharmaceutical agents in the year of diagnosis of CVD, and health care expenses was used to calculate the inverse propensity score. The crude hazard ratio (HR) (with 95% CI) was calculated by univariable Cox proportional hazards models. Subsequently, we corrected for sex, age, category of CVD diagnosis, cardiothoracic surgery, Charlson Comorbidity Index, and cardiac medication. Stabilized inverse propensity score weighting was applied to the fully adjusted multivariable Cox proportional hazards models to account for differences between CR participants and nonparticipants.^24,25,26^ We evaluated the robustness of the main outcomes via sensitivity analyses. For this purpose, the period between the initial event date and the minimum follow-up period was changed from 180 days to 1 to 6 years.

The presence of effect modification was tested for using an interaction term to assess the implications of sex (reference group, men), age (reference group, <50 years), SES (reference group, low SES), CVD diagnosis (reference group, chronic heart failure), cardiothoracic surgery (reference group, elective PCI), and comorbidity (reference group, CCI≤1) for the association between CR participation and all-cause mortality risk. Two-sided *P* < .05 was considered statistically significant. All statistical analyses were performed in SAS, version 9.4 (SAS Institute Inc).

## Results

### Patient Characteristics

**Figure 1.**
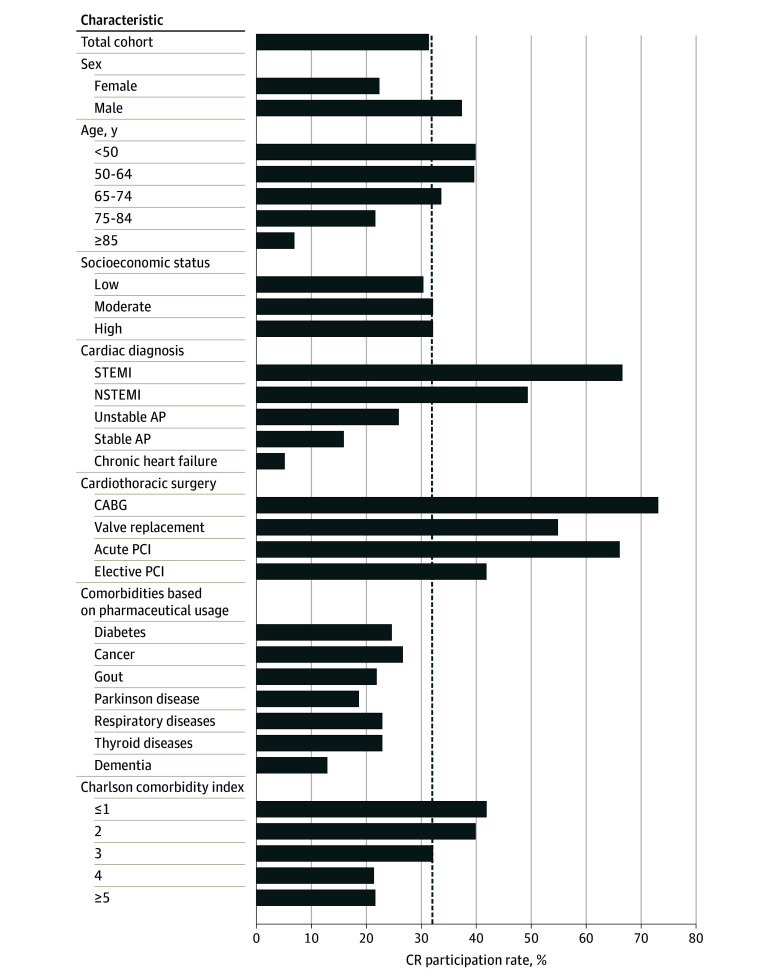
Cardiac Rehabilitation (CR) Participation Rates Across Strata of Patient and Disease Characteristics The dashed line represents the average CR participation rate (31.3%) of the analytic cohort (N = 83 687). AP indicates angina pectoris; CABG, coronary artery bypass grafting; NSTEMI, non–ST-segment elevation myocardial infarction; PCI, percutaneous coronary intervention; and STEMI, ST-segment elevation myocardial infarction.

In this cohort of 83 687 patients with CVD (mean [SD] age, 67 [12] years; 60.4% [n = 50 512] men), only 31.3% (n = 26 171) participated in CR at 89 CR facilities in the Netherlands. The CR participation rate increased across calendar years from 25.7% in 2012 to 38.6% in 2017 (eTable 1 in the Supplement). Distance to the CR facility was greater for CR participants than for nonparticipants (mean [SD], 15.4 [19.3] vs 14.7 [20.2] km; *P* < .001). Compared with nonparticipants, CR participants were younger, included more male patients, had a lower CCI score, and had a higher income (Table 1). Cardiac medication use was lower in the year before CVD diagnosis among CR participants compared with nonparticipants, but this association largely reversed in the year after CVD diagnosis. Cardiac rehabilitation participation rates were higher among men (37.2%), patients younger than 75 years (33.5% to 39.8%), those with a diagnosis of STEMI (66.6%) or NSTEMI (49.2%), those with a CCI score of 3 or less (32.0% to 41.7%), and those undergoing CABG (73.0%), valve replacement (54.8%), or acute (66.0%) or elective (41.8%) PCI. In contrast, CR was underused in women (22.2%), patients 75 years or older (6.7% to 21.5%), those with comorbidity (12.7% to 26.5%), and those having a CVD diagnosis of unstable (25.9%) or stable (15.9%) AP or chronic heart failure (5.1%) (Figure 1).

**Table 1.** Comparison of Patient Characteristics Between Cardiac Rehabilitation (CR) Participants and Nonparticipants

Variable	No. (%)		*P* value
	CR participants (n = 26 171)	Nonparticipants (n = 57 516)	
Male sex	18 790 (71.8)	31 722 (55.2)	<.001
Age, mean (SD), y	64 (11)	69 (12)	<.001
CCI in the year before the study, mean (SD)	3.1 (3.8)	4.4 (6.6)	<.001
Income, mean (SD), €	36 000 (7000)^a^	35 500 (7200)^b^	<.001
SES			<.001
Low	11 188 (42.7)	25 656 (44.6)	
Moderate	8233 (31.5)	17 526 (30.5)	
High	6750 (25.8)	14 334 (24.9)	
Distance to CR facility, km	15.4 (19.3)	14.7 (20.2)	<.001
CVD diagnosis			
STEMI	8169 (31.2)	4096 (7.1)	<.001
NSTEMI	7881 (30.2)	8123 (14.2)	
Unstable AP	3571 (13.6)	10 242 (17.8)	
Stable AP	3897 (14.9)	20 652 (35.9)	
Chronic heart failure	495 (1.9)	9285 (16.1)	
Only cardiothoracic surgery	2158 (8.2)	5118 (8.9)	
Cardiothoracic surgery			
CABG	4078 (15.6)	1511 (2.6)	<.001
Valve replacement	1327 (5.1)	1095 (1.9)	
Acute PCI	10 396 (39.7)	5350 (9.3)	
Elective PCI	5908 (22.6)	8235 (14.3)	
No cardiothoracic surgery	4462 (17.0)	41 325 (71.9)	
CCI score			
≤1	7210 (27.5)	10 078 (17.5)	<.001
2	6720 (25.7)	10 195 (17.7)	
3	4976 (19.0)	10 580 (18.4)	
4	2516 (9.6)	9315 (16.2)	
≥5	4749 (18.1)	17 348 (30.2)	
Cardiac medication use in the year before CVD diagnosis			
ACE inhibitors	5553 (21.1)	17 577 (30.6)	<.001
Angiotensin II receptor blockers	4648 (17.8)	13 303 (23.1)	<.001
Antithrombotic drugs	11 654 (44.5)	37 830 (65.8)	<.001
β-Blockers	9879 (37.7)	31 610 (55.0)	<.001
Calcium channel blockers	4939 (18.9)	14 607 (25.4)	<.001
Diuretics	11 151 (42.6)	31 429 (54.6)	<.001
Lipid-lowering drugs	4789 (18.3)	20 155 (35.0)	<.001
Other antihypertensive agents	313 (1.2)	1172 (2.0)	<.001
Cardiac medication in the year after CVD diagnosis			
ACE inhibitors	17 625 (67.3)	25 772 (44.8)	<.001
Angiotensin II receptor blockers	6308 (24.1)	14 423 (25.1)	.003
Antithrombotic drugs	26 061 (99.6)	51 156 (88.9)	<.001
β-Blockers	23 747 (90.7)	44 186 (76.8)	<.001
Calcium channel blockers	7559 (28.9)	18 184 (31.6)	<.001
Diuretics	7975 (30.5)	25 206 (43.8)	<.001
Lipid-lowering drugs	24 896 (95.1)	42 994 (74.8)	<.001
Other antihypertensive agents	411 (1.6)	1424 (2.5)	<.001

Abbreviations: ACE, angiotensin-converting enzyme; AP, angina pectoris; CABG, coronary artery bypass grafting; CCI, Charlson Comorbidity Index; CVD, cardiovascular disease; NSTEMI, non–ST-segment elevation myocardial infarction; PCI, percutaneous coronary intervention; SES, socioeconomic status; STEMI, ST-segment elevation myocardial infarction.

^a^ US $40 475 ($7870).

^b^ US $39 914 ($8095).

### Clinical Outcomes

**Figure 2.**
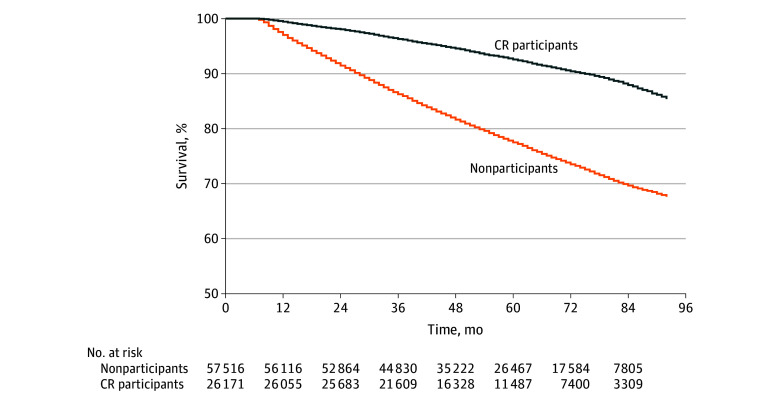
Kaplan-Meier Curves of All-Cause Mortality During Follow-up Cardiac rehabilitation (CR) participation was associated with statistically significantly better event-free survival compared with nonparticipation (log-rank test comparing curves, *P* < .001 for overall survival).

**Figure 3.**
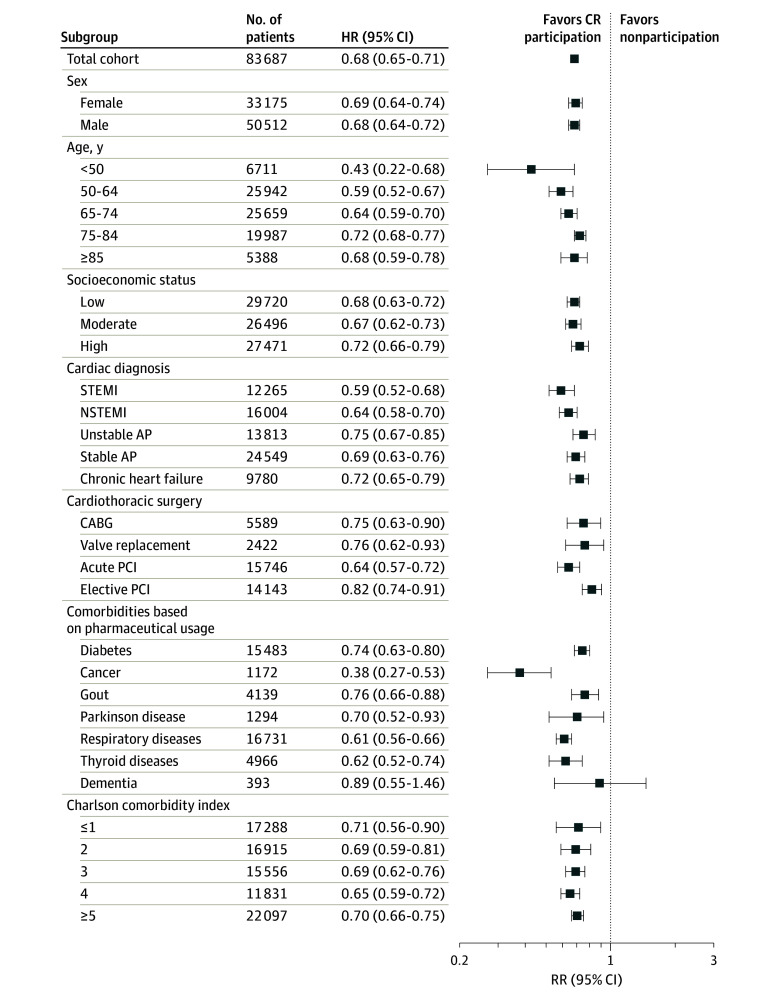
Subgroup-Specific Adjusted Hazard Ratios (HR) for All-Cause Mortality Risk Among Patients With Cardiovascular Disease Eligible to Enroll in a Cardiac Rehabilitation (CR) Program Data are presented on a logarithmic scale. A 32% lower risk of all-cause mortality was found for the total cohort, and adjusted HRs between 0.38 (95% CI, 0.27-0.53) and 0.89 (95% CI, 0.55-1.46) were observed across subgroups. Participation in CR is favored for all subgroups because risk estimates were all less than 1. However, a nonstatistically significant risk reduction was observed for patients with dementia likely because of the small sample size, contributing to a wide 95% CI. AP indicates angina pectoris; CABG, coronary artery bypass grafting; NSTEMI, non–ST-segment elevation myocardial infarction; PCI, percutaneous coronary intervention; and STEMI, non–ST-segment elevation myocardial infarction.

During a mean (SD) of 4.7 (1.8) years of follow-up (56 [22] months), 1966 CR participants (7.5%) and 13 443 nonparticipants (23.4%) died (*P* < .001) (Table 2). Kaplan-Meier analysis revealed that CR participation was associated with better event-free survival (Figure 2). Cox proportional hazards models showed a 68% lower risk of all-cause mortality (crude HR, 0.32; 95% CI, 0.30-0.33) in CR participants compared with nonparticipants (Table 2). Mortality risk remained lower in CR participants after stepwise correction for confounders (adjusted HR, 0.68; 95% CI, 0.65-0.71) (eTable 2 in the Supplement). After multivariable adjustment, CR participation was associated with a 32% lower risk of all-cause mortality (adjusted HR, 0.68; 95% CI, 0.65-0.71) compared with nonparticipation. Mortality rates during follow-up were variable across subgroups (Table 2), ranging from 1.3% to 63.9%. Cardiac rehabilitation participation was associated with improved event-free survival and a statistically significantly lower risk of all-cause mortality among all subgroups, except for patients with dementia (Figure 3). Sex, age, SES, and comorbidity did not alter risk reduction after CR participation, with no effect modification observed. However, a statistically significant interaction association was found across categories of CVD diagnosis and cardiothoracic surgery. Larger reductions in risk estimates for all-cause mortality were found after CR participation for STEMI (adjusted HR, 0.59; 95% CI, 0.52-0.68 vs 0.72; 95% CI, 0.65-0.79; *P* < .001), NSTEMI (adjusted HR, 0.64; 95% CI, 0.58-0.70 vs 0.72; 95% CI, 0.65-0.79; *P* < .001), and stable AP (adjusted HR, 0.69; 95% CI, 0.63-0.76 vs 0.72; 95% CI, 0.65-0.79; *P* < .001) compared with patients with chronic heart failure, whereas unstable AP had a smaller risk reduction (adjusted HR, 0.75; 95% CI, 0.67-0.85 vs 0.72; 95% CI, 0.65-0.79; *P* < .001). Effect modification was also present for cardiothoracic surgery, with acute PCI having a larger reduction in the risk estimate for all-cause mortality after CR participation (adjusted HR, 0.64; 95% CI, 0.57-0.72 vs 0.82; 95% CI, 0.74-0.91; *P* < .001) compared with elective PCI, whereas no difference in risk reduction was found for CABG (adjusted HR, 0.75; 95% CI, 0.63-0.90 vs 0.82; 95% CI, 0.74-0.91; *P* = .53) or valve replacement (adjusted HR, 0.76; 95% CI, 0.62-0.93 vs 0.82; 95% CI, 0.74-0.91; *P* = .53).

**Table 2.** Mortality Rates and Crude Hazard Ratios (HR) for the Total Cohort and Subgroups

Variable	Total cohort, No.	CR participants			Nonparticipants			Crude HR (95% CI)
		No.	Deaths, No. (%)	Deaths per 1000 person-years	No.	Deaths, No. (%)	Deaths per 1000 person-years	
Total cohort	83 687	26 171	1966 (7.5)	15.9	57 516	13 443 (23.4)	50.3	0.32 (0.30-0.33)
Sex								
Female	33 175	7381	541 (7.3)	15.6	25 794	5903 (22.9)	49.4	0.32 (0.29-0.34)
Male	50 512	18 790	1425 (7.6)	16.0	31 722	7540 (23.8)	51.1	0.31 (0.30-0.33)
Age, y								
<50	6711	2668	36 (1.3)	2.7	4043	135 (3.3)	6.3	0.43 (0.30-0.63)
50-64	25 942	10 243	357 (3.5)	7.2	15 699	1201 (7.7)	14.9	0.49 (0.43-0.55)
65-74	25 659	8605	667 (7.8)	16.4	17 054	2986 (17.5)	36.5	0.45 (0.42-0.49)
75-84	19 987	4294	788 (18.4)	41.5	15 693	5907 (37.6)	89.1	0.46 (0.43-0.50)
≥85	5388	361	118 (32.7)	84.6	5027	3214 (63.9)	193.1	0.42 (0.35-0.51)
SES								
Low	36 844	11 188	900 (8.0)	17.0	25 656	6383 (24.9)	54.1	0.31 (0.29-0.34)
Moderate	25 759	8233	622 (7.6)	16.0	17 526	4086 (23.3)	50.2	0.32 (0.29-0.35)
High	21 084	6750	444 (6.6)	13.9	14 334	2974 (20.7)	43.9	0.32 (0.29-0.35)
CVD diagnosis								
STEMI	12 265	8169	488 (6.0)	12.6	4096	917 (22.4)	47.8	0.26 (0.24-0.29)
NSTEMI	16 004	7881	595 (7.5)	16.4	8123	2460 (30.3)	70.2	0.23 (0.21-0.25)
Unstable AP	13 813	3571	284 (8.0)	16.1	10 242	1819 (17.8)	35.1	0.46 (0.41-0.52)
Stable AP	24 549	3897	265 (6.8)	14.3	20 652	2506 (12.1)	24.4	0.60 (0.53-0.68)
Chronic heart failure	9780	495	97 (19.6)	46.2	9285	4263 (45.9)	119.7	0.38 (0.31-0.47)
Cardiothoracic surgery								
CABG	5589	4078	313 (7.7)	15.6	1511	232 (15.4)	30.0	0.53 (0.45-0.63)
Valve replacement	2422	1327	164 (12.4)	25.7	1095	276 (25.2)	54.1	0.48 (0.39-0.57)
Acute PCI	15 746	10 396	606 (5.8)	12.5	5350	1065 (19.9)	42.6	0.29 (0.27-0.33)
Elective PCI	14 143	5908	465 (7.9)	16.8	8235	1367 (16.6)	34.7	0.49 (0.44-0.54)
Comorbidity based on pharmaceutical agent use								
Diabetes	15 483	3788	511 (13.5)	29.5	11 695	3970 (33.9)	78.7	0.37 (0.34-0.41)
Cancer	1172	311	39 (12.5)	28.3	861	290 (33.7)	81.7	0.34 (0.25-0.48)
Gout	4139	901	142 (15.8)	34.9	3238	1326 (41.0)	100.9	0.34 (0.29-0.41)
Parkinson disease	1294	240	33 (13.8)	29.2	1054	396 (37.6)	87.9	0.33 (0.23-0.47)
Respiratory diseases	16 731	3807	431 (11.3)	24.5	12 924	4430 (34.3)	79.9	0.30 (0.28-0.34)
Thyroid diseases	4966	1131	109 (9.6)	20.6	3835	1055 (27.5)	61.4	0.33 (0.27-0.41)
Dementia	393	50	10 (20.0)	46.4	343	204 (59.5)	153.2	0.30 (0.16-0.56)
CCI								
≤1	17 288	7210	144 (2.0)	4.1	10 078	293 (2.9)	5.5	0.75 (0.62-0.72)
2	16 915	6720	243 (3.6)	7.5	10 195	688 (6.7)	13.1	0.58 (0.50-0.67)
3	15 556	4976	415 (8.3)	17.8	10 580	1876 (17.7)	36.1	0.51 (0.45-0.56)
4	11 831	2516	363 (14.4)	31.3	9315	3409 (36.6)	84.8	0.36 (0.33-0.41)
≥5	22 097	4749	801 (16.9)	38.5	17 348	7177 (41.4)	104.1	0.37 (0.34-0.39)

Abbreviations: AP, angina pectoris; CABG, coronary artery bypass grafting; CCI, Charlson Comorbidity Index; CR, cardiac rehabilitation; CVD, cardiovascular disease; NSTEMI, non–ST-segment elevation myocardial infarction; PCI, percutaneous coronary intervention; SES, socioeconomic status; STEMI, ST-segment elevation myocardial infarction.

To evaluate the robustness of the main outcomes, the adjusted HRs of CR participation on all-cause mortality were recalculated. For this purpose, mortality risk reduction for different durations during follow-up was calculated. These sensitivity analyses showed risk reduction for all-cause mortality similar to the initial findings (adjusted HR, 0.68-0.86) (eTable 3 in the Supplement).

## Discussion

Cardiac rehabilitation programs aim to improve prognosis and quality of life via a multifaceted intervention. The present study demonstrated that CR participation was associated with a 32% lower risk of all-cause mortality compared with nonparticipation after adjustment for confounding factors. The association between CR participation and all-cause mortality was independent of sex, age, socioeconomic status, and comorbidity, but the magnitude of risk reduction after CR differed across categories of CVD diagnosis and type of cardiothoracic surgery. On average, the CR participation rate was 31.3%, but it increased from 25.7% in 2012 to 38.6% in 2017. Large variation in CR use was observed (range, 5.1%-73.0%) across different subgroups. Findings from this Dutch nationwide observational cohort study underscore the health benefit of CR and emphasize the need to better use CR, such as improving referral and encouragement by clinicians, particularly in vulnerable patients with CVD (eg, older individuals or those with comorbidity or multimorbidity).

### All-Cause Mortality

The crude risk reduction in all-cause mortality after CR participation was 68%, which was reduced to 32% after adjustment for confounding factors. The quantification of mortality risk reduction in the present study is substantially larger compared with outcomes of previously published meta-analyses (−1% to 13%).^2,5,27,28^ A potential explanation for the discrepant outcomes may relate to the study design. We used an observational population-based cohort to study the health benefit of CR in a real-world setting, yielding high external validity. This design is in contrast to meta-analyses, which use data from randomized clinical trials (RCTs). Although RCTs may reduce the risk of confounding because of randomization, vulnerable patients (eg, older individuals or those with comorbidity or multimorbidity) are typically excluded. Hence, RCTs have high internal validity but typically have lower external validity.^29^ The health benefit of CR may be larger in patients who are underrepresented in or excluded from RCTs. Alternatively, the content of the CR program may alter the outcomes.^17^ Our patients were enrolled in a multidisciplinary program, including supervised exercise training, mental health and stress relief, social health, and cardiovascular risk management,^16^ whereas most RCTs compared exercise-based CR with a control condition.^5^ Hence, mortality risk reduction may have been larger in our cohort because of the multifaceted treatment that patients received.^30^ Indeed, our findings align with other large observational population studies^20,31,32^ reporting a 33% to 35% risk reduction in CR participants. Independent of the risk reduction of the present study vs data from RCTs, these joint outcomes further reinforce the Class IA recommendation for CR participation in current and future clinical guidelines of professional associations.^7,8,9,10^

### Risk Reduction Across Subgroups

A unique aspect of the present study is the assessment of mortality risk across different subgroups of patients eligible for CR. Better survival was found for all subgroups participating in CR. The magnitude of risk reduction after CR differed statistically significantly across categories of CVD diagnosis and type of cardiothoracic surgery, but risk estimates and their 95% CIs were less than 1 for every subgroup (Figure 3), indicating that CR participation provides a better outcome than nonparticipation. This finding is notable because the level of evidence of CR health benefits is weak for some subgroups. Few studies on CR report sex-specific outcomes,^33^ but risk reduction after CR differs between men and women.^20^ Although CR use is lower in women than in men, we observed a similar mortality risk reduction with CR participation in men (32.1%) and women (31.3%) (Figure 3), suggesting that both sexes benefit equally from CR.

Older patients are often underrepresented in CR programs, despite their higher disease prevalence.^34^ Hence, recommendations for treatment of older patients (≥75 years) are needed that are typical of those individuals encountered in routine clinical practice.^35^ Age did not alter the association between CR participation and better mortality outcomes. In fact, CR was associated with large reductions in all-cause mortality in patients aged 75 to 84 years (27.8%) and 85 years or older (32.0%) (Figure 3). These findings suggest that CR improves survival even in older age groups, which have mortality rates of up to 63.9%.

The implications of CR for mortality in patients with stable AP remain unclear. In a recent systematic review and meta-analysis^28^ (including 7 studies and 581 patients), risk estimates could not be calculated because of insufficient power. Among 24 549 patients with stable AP in our study, CR participation was associated with a 31% mortality risk reduction (adjusted HR, 0.69; 95% CI, 0.63-0.77) (Figure 3). Similarly, among 13 813 patients with unstable AP, CR participation was associated with a 25% mortality risk reduction (adjusted HR, 0.75; 95% CI, 0.67-0.85). Our observations underscore the mortality benefit of CR for patients with stable AP and patients with unstable AP.

Multiple comorbid conditions are increasingly prevalent among patients with CVD.^36^ Patients with multimorbidity have a worse prognosis^37^ and a lower CR participation rate,^38^ and current clinical practice is mainly targeted toward care of patients with a single disease. We found that CR participants with diabetes, cancer, gout, Parkinson disease, respiratory diseases, and thyroid diseases had better survival rates compared with nonparticipants. Patients with CVD with dementia did not demonstrate a statistically significant risk reduction after CR, but the risk estimate indicated a 10.8% reduction in all-cause mortality (adjusted HR, 0.89; 95% CI, 0.55-1.46) (Figure 3). The lack of statistical significance is likely because of the small sample size (n = 393), which contributed to a wide 95% CI (−45% to 46%). Cardiac rehabilitation participation among patients with CVD with the highest CCI scores was associated with notable risk reduction (≥30%). These findings emphasize that CR has substantial health value for patients with CVD with comorbidity and should lead clinicians to encourage their patients to participate in CR.

### CR Use

Findings from the present study not only reinforce the health benefit of CR but also demonstrate the underuse of this powerful intervention. Only 31.3% of eligible Dutch patients participated in CR, which is high compared with US data^11,12^ but is similar to values reported in European studies.^39,40^ However, more notable is the increase in CR use from 25.7% in 2012 to 38.6% in 2017. Future studies are warranted to elucidate the reasons for this substantial improvement as well as to understand temporal changes in predictors of participation. Outcomes from such analyses can be used to develop strategies to further increase CR use across the globe.

Large variability in CR participation rates across different subgroups was also observed. Participation rates were high for medical conditions with clear guidelines and strong recommendations, such as STEMI^9^ (66.6%), CABG^7^ (73.0%), valve replacement^7^ (54.8%), and acute (66.0%) or elective (41.8%) PCI.^41^ Participation rates were low in subgroups with chronic conditions, such as older patients and those with stable AP, chronic heart failure, comorbidities (ie, diabetes, cancer, gout, etc), and multimorbidity (CCI≥4) (Figure 1). The lower participation rates likely relate to the frailty of these patients, a lack of physician referral, or to practical barriers, such as a lack of transportation.^42^ Alternative strategies to deliver CR, such as home-based CR,^43,44^ telerehabilitation,^45^ or eHealth interventions,^46^ may enable patients with CVD to profit from CR.

### Limitations

This study has some limitations. The main limitation is its observational design. Analyses based on CR components were not possible to perform because of a change in the medical claims registry system during the study period and an insufficient level of detail regarding the number of sessions and the combination of components. The use of health insurance claims data did not allow us to control for all potential confounding factors, such as cardiovascular risk factors, disease severity, lifestyle (eg, physical activity, smoking, and diet), and patient motivation. Nevertheless, we were able to extract data regarding patient characteristics, CVD diagnosis, type of cardiothoracic surgery, hospital stay, pharmaceutical agent use, comorbidity, and health care costs to correct for differences between CR participants and nonparticipants. Stabilized inverse propensity score weighting was used to create a pseudopopulation and subsequently adjust for the covariates in the Cox proportional hazards models to minimize potential group differences. Matched-pairs analysis (n = 32 780) confirmed the findings of our primary analysis (data not shown). Race was not available, but most of the Dutch population is of white race/ethnicity (approximately 87.7%).^47^ Residual confounding may have occurred because unmeasured variables could explain some of the observed mortality differences between CR participants and nonparticipants. However, results from the present study demonstrated a consistent mortality risk reduction among all subgroups of CR participants, and the health benefit of CR was further reinforced in the sensitivity analyses. Finally, the inclusion of a large and heterogeneous patient population in combination with the high accuracy of health insurance data^48^ supports the value of the present study beyond previously published RCTs and meta-analyses.

## Conclusions

After multivariable adjustment, participation in a multidisciplinary CR program was associated with 32% lower risk of all-cause mortality (adjusted HR, 0.68; 95% CI, 0.65-0.71) compared with nonparticipation during a mean (SD) of 4.7 (1.8) years of follow-up. The survival benefit associated with CR participation was observed in virtually all subgroups of patients with CVD (except for patients with dementia), including risk estimates specific to sex, age, SES, CVD diagnosis, cardiothoracic surgery, and comorbidity. Findings from the present study further reinforce current recommendations of the American Heart Association, American College of Cardiology, and European Society of Cardiology and suggest that CR should be prescribed more widely to vulnerable patients with CVD, such as older adults with chronic diseases or multimorbidity. Cardiac rehabilitation participation rates remain low in these at-risk subgroups, whereas the benefits of cardiac rehabilitation are similar to those of low-risk subgroups.

## Notes

Supplement. **eTable 1.** Yearly Inclusion Rate of Patients Eligible for CR Participation Among the VGZ Cohort **eTable 2.** Crude and Multivariable Adjusted Hazard Ratios for All-Cause Mortality Among CR Participants **eTable 3.** Sensitivity Analyses for Different Thresholds of Minimal Follow-up Time **eFigure 1.** The VGZ Cohort Is a Valid Representation of the Dutch Population **eFigure 2.** Flowchart of Patient Enrollment From the VGZ Health Insurance Database
