# Supplementary material for: Association of Cardiac Rehabilitation With All-Cause Mortality Among Patients With Cardiovascular Disease in the Netherlands
Source: JAMA Netw Open. 2020 Jul 27;3(7):e2011686. doi: 10.1001/jamanetworkopen.2020.11686 (PMC12124693; doi:10.1001/jamanetworkopen.2020.11686)
Supplement: Supplement. — eTable 1. Yearly Inclusion Rate of Patients Eligible for CR Participation Among the VGZ Cohort eTable 2. Crude and Multivariable Adjusted Hazard Ratios for All-Cause Mortality Among CR Participants eTable 3. Sensitivity Analyses for Different Thresholds of Minimal Follow-up Time eFigure 1. The VGZ Cohort Is a Valid Representation of the Dutch Population eFigure 2. Flowchart of Patient Enrollment From the VGZ Health Insurance Database [file jamanetwopen-e2011686-s001.pdf]

Supplementary Online Content

Eijsvogels TMH, Maessen MFH, Bakker EA, et al. Association of cardiac rehabilitation with all-cause mortality among patients with cardiovascular disease in the Netherlands. *JAMA Netw Open*. 2020;3(7):e2011686. doi:10.1001/jamanetworkopen.2020.11686

eTable 1. Yearly Inclusion Rate of Patients Eligible for CR Participation Among the VGZ Cohort

eTable 2. Crude and Multivariable Adjusted Hazard Ratios for All-Cause Mortality Among CR Participants

eTable 3. Sensitivity Analyses for Different Thresholds of Minimal Follow-up Time

eFigure 1. The VGZ Cohort Is a Valid Representation of the Dutch Population

eFigure 2. Flowchart of Patient Enrollment From the VGZ Health Insurance Database

This supplementary material has been provided by the authors to give readers additional information about their work.

© 2020 Eijsvogels TMH et al. *JAMA Network Open*.

eTable 1. Yearly Inclusion Rate of Patients Eligible for CR Participation Among the VGZ Cohort

Inclusion year	Total sample *(n)*	CR-participants *(n (%))*	CR-participants Deaths (n (%))	Non-participants *(n (%))*	Non-participants Deaths (n (%))
2012	10,050	2,582 (25.7)	388 (15.0)	7,468 (74.3)	2,345 (31.4)
2013	17,452	4,354 (24.9)	446 (10.2)	13,098 (75.1)	3,842 (29.3)
2014	15,106	4,155 (27.5)	342 (8.2)	10,951 (72.5)	2,770 (25.3)
2015	13,886	4,791 (34.5)	382 (8.0)	9,095 (65.5)	1,970 (21.7)
2016	13,805	5,125 (37.1)	251 (4.9)	8,680 (62.9)	1,507 (17.4)
2017	13,388	5,164 (38.6)	157 (3.0)	8,224 (61.4)	1,009 (12.3)

© 2020 Eijsvogels TMH et al. *JAMA Network Open*.

eTable 2. Crude and Multivariable Adjusted Hazard Ratios for All-Cause Mortality Among CR Participants. A total of 83,687 patients was included in this study, of which 26,171 CR-participants (1,966 deaths, 7.5%) and 57,516 non-participants (13,443 deaths, 23.4%).

Model	Hazard Ratio	95% Confidence Interval
**Model 1** [a]	0.32	0.30 - 0.33
**Model 2** [b]	0.46	0.44 - 0.49
**Model 3** [c]	0.52	0.49 - 0.55
**Model 4** [d]	0.56	0.53 - 0.59
**Model 5** [e]	0.68	0.65 - 0.71

[a] crude model; [b] model 1 plus sex and age; [c] model 2 plus CVD diagnosis, intervention and hospital stay; [d] model 3 plus charlson comorbidity index and cardiovascular pharmaceuticals; [e] model 4 plus propensity weighted adjustment.

© 2020 Eijsvogels TMH et al. *JAMA Network Open*.

eTable 3. Sensitivity Analyses for Different Thresholds of Minimal Follow-up Time.

Minimal follow-up time (years)	Total cohort	Cardiac rehab participants		Non-participants		Hazard ratio (crude)	Hazard ratio (adjusted)
	n	n	Deaths, n (%)	n	Deaths, n (%)		
<1	83,687	26,171	1,966 (7.5)	57,516	13,443 (23.4)	0.32 (0.30-0.33)	0.68 (0.65-0.71)
1	82,171	26,055	1,850 (7.1)	56,116	12,043 (21.5)	0.33 (0.31-0.35)	0.68 (0.65-0.72)
2	78,547	25,683	1,478 (5.8)	52,864	8,791 (16.6)	0.36 (0.34-0.39)	0.70 (0.66-0.74)
3	66,439	21,609	1,061 (4.9)	44,830	5,851 (13.1)	0.40 (0.38-0.43)	0.71 (0.67-0.76)
4	51,550	16,328	715 (4.4)	35,222	3,659 (10.4)	0.45 (0.42-0.49)	0.76 (0.71-0.83)
5	37,954	11,487	422 (3.7)	26,467	2,032 (7.7)	0.49 (0.44-0.55)	0.79 (0.72-0.88)
6	24,984	7,400	197 (2.7)	17,584	850 (4.8)	0.55 (0.47-0.64)	0.86 (0.74-1.00)

© 2020 Eijsvogels TMH et al. *JAMA Network Open*.

eFigure 1. The VGZ Cohort (blue bars, 4.1 million individuals) is a valid representation of the Dutch population (orange bars, 17.3 million individuals) as the relative contribution of A) age categories, B) sex distribution, and C) socioeconomic status, were comparable between both cohorts.

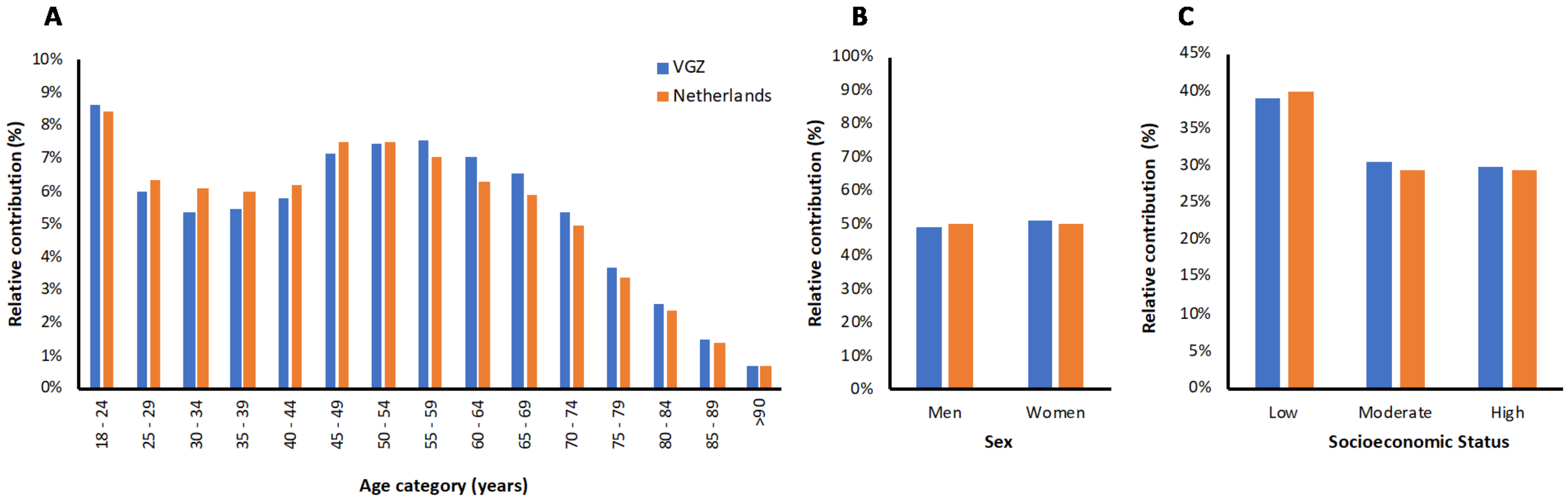

© 2020 Eijsvogels TMH et al. *JAMA Network Open*.

eFigure 2. Flowchart of patient enrollment from the VGZ Health Insurance Database.

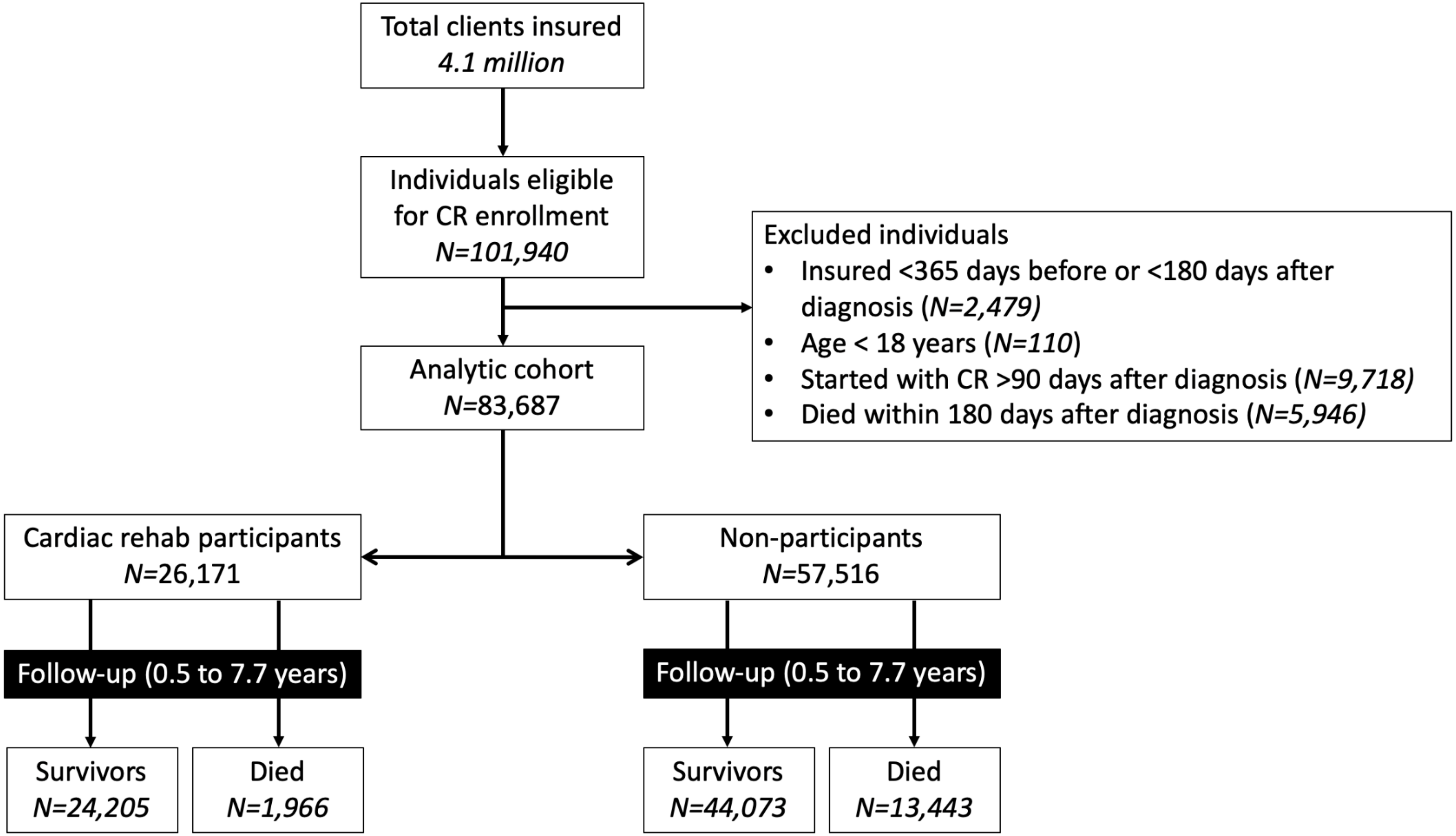

© 2020 Eijsvogels TMH et al. *JAMA Network Open*.
